# Challenges and Opportunities in the Supply of Living Kidney Donation in the UK National Health Service: An Economic Perspective

**DOI:** 10.1097/TP.0000000000004176

**Published:** 2022-05-27

**Authors:** Tiyi Morris, Hannah Maple, Sam Norton, Joseph Chilcot, Lisa Burnapp, Heather Draper, Nizam Mamode, Paul McCrone

**Affiliations:** 1 King’s Health Economics, Health Service and Population Research Department, Institute of Psychiatry, Psychology and Neuroscience, King’s College London, London, United Kingdom.; 2 Department of Applied Health Research, University College London, London, United Kingdom.; 3 Department of Renal Transplantation, Guy’s and St. Thomas’ NHS Foundation Trust/King’s College London, London, United Kingdom.; 4 Department of Psychology, Institute of Psychiatry, Psychology and Neuroscience, King’s College London, London, United Kingdom.; 5 Directorate of Organ and Tissue Donation and Transplantation, NHS Blood and Transplant, Bristol, United Kingdom.; 6 Division of Health Sciences, Warwick Medical School, University of Warwick, Coventry, United Kingdom.; 7 Institute for Lifecourse Development, University of Greenwich, London, United Kingdom.

## Abstract

End-stage kidney disease is a significant burden on the healthcare systems of many countries, and this is likely to continue because of an increasingly aging and comorbid population. Multiple studies have demonstrated a significant clinical benefit in transplantation when compared with dialysis, however, there continues to be a shortage of donor kidneys available. This article provides an economic perspective on issues pertinent to living kidney donation and transplantation. Although ethics, equity, and cultural considerations often seem at odds with economic concepts around resource allocation, this article explains the situation around supply and demand for living kidneys and illustrates how this has been addressed in the economic literature. The article discusses different policy recommendations for resolving the imbalance between supply and demand in kidney donation, through policies under 3 main approaches: increasing supply, decreasing demand, and improving the allocation of kidney supply.

## INTRODUCTION

The projected number of people with chronic kidney disease (CKD) in England is expected to rise, from 2 623 504 in 2011 to 4 199 203 by 2036, affecting around 8.3% of the population.^1^ This is predominantly because of an increase in age and comorbidity and is based on the assumption that treatments for end-stage kidney disease (ESKD) will not dramatically improve. This is likely to be an issue in other countries with aging populations and high prevalence of comorbidity including diabetes and hypertension. In addition to the direct impact on patients, treatment for CKD and ESKD comes at a cost to the economy. In England, it accounted for around 1.3% of the National Health Service (NHS) budget in 2009–2010, then totaling an estimated £1.44–£1.45 billion.^2^ This is set to increase in proportion to the increase in older people in the population. Healthcare requirements will generally increase as the population ages and so establishing cost-effective approaches as well as increasing resource availability is required.

There are significant costs attached to all treatment modalities in kidney disease. The annual cost of hemodialysis is in the order of £37 500 per annum per patient, derived from Pyart et al.^3^ Studies have shown that kidney transplantation, despite the incurred costs of surgery for both the donor and recipient and the need for immunosuppressive medication for the duration of the transplant is more cost-effective than dialysis.^4-6^ We discuss the UK context in particular, because of the organization of the NHS as free at the point of delivery, and the progress that has been made in unspecified living kidney transplantation.^7^

Economics is the study of how society allocates resources when there is scarcity, and health economics addresses the efficiency and equity of how this is done when providing healthcare.^8^ Kidney transplantation is a unique case because the good is a human organ and there are ethical implications about the way we treat other human beings and their bodies, but it is also a supply and demand problem like many in health (in universal healthcare systems) in which no transfers or prices are present.^9,10^ As well as efficiency in the relationship between supply and demand, there are also considerations to be made around equity particularly around access to treatment for ethnic minority populations in the United Kingdom.^11^

This article outlines some of the key issues surrounding the supply of and demand for kidneys for people with ESKD. It does this from an economic perspective and investigates whether mechanisms by which supply, and demand are modified elsewhere could be applied in this clinical area. The study has arisen from the BOUnD project, which is investigating nonspecified living donation. The BOUnD study is about the impact of unspecified living kidney donations and proposes the hypothesis that there are no negative impacts of living kidney donation on physical or mental health.^12^ Arguments in favor of using these donors are that this (a) creates additional transplant opportunities for patients, with or without a living donor of their own (via the waiting list or by priming paired exchange chains) and (b) helps transplant immunologically complex patients and patients who have waited a long time on the waiting list, thereby reducing the economic burden further.

## MATERIALS AND METHODS

We reviewed MEDLINE, EMBASE, and Google Scholar databases.^13-15^ We also discussed the article with experts on transplant surgery, kidney donation, psychology around donation, and medical ethics within the team. We aimed to understand the issues around supply and demand of living kidneys for donation and propose policies and solutions to improve the situation in the United Kingdom. Our recommendations are UK system-specific, because of context having such an impact on what is ethically, politically, and economically acceptable, even though we do consider examples from around the world.

## SUPPLY AND DEMAND ISSUES AROUND KIDNEY DONATION

By December 2017, over 64 000 patients in the United Kingdom were being treated for ESKD, 55% of whom were transplant recipients (UK Renal Registry Report, 2018).^16^ Since becoming an established practice, transplantation has been affected by an imbalance between supply and demand, with many more people requiring a transplant than can be met by current donations from both living and deceased donors. As of January 1, 2020, 4825 people were on the waiting list for a kidney transplant, and 200 people with diabetes and renal failure were awaiting a combined kidney and pancreas transplant.^17^

Although there are still a considerable number of people awaiting a kidney transplant, the waiting list has reduced significantly over recent years until the COVID-19 pandemic. Over 7000 people were on the waiting list for a kidney at the beginning of the last decade, and the increase in transplant numbers can be attributed to an increase in both living and deceased donor numbers (NHS Blood and Transplant, 2019).^7^

We are not alone in considering kidney donation as a problem of supply and demand. Testa and Siegler^18^ wrote that there is a supply and demand imbalance in the United States and Europe, *The Economist*^19^ wrote in 2008 that the idea of paying donors is gaining support, and Agarwal et al^20^ explains how the concept of supply and demand can be applied in markets in which there are no prices. The unique contribution of this review is to assess how this problem occurs in the UK context and in the case of living kidney donation.

The discrepancy between organ supply and demand has been influenced by advances in modern medicine, which have led to more patients surviving catastrophic events or injuries that would otherwise have killed them (such as strokes and road traffic accidents) and more patients being eligible for transplantation who previously may not have been. These include those who have received transplants previously, those with complex anatomy, and those who have other comorbidities that would have previously precluded transplantation. Organizational and logistical changes implemented by the NHS have increased the number of deceased donors, but living donation has also greatly increased. The increased utilization of organs from those in whom death is diagnosed by cessation of circulatory criteria (donation after circulatory death [DCD]) has also contributed to improved supply.^21^ There was previously a degree of hesitancy in utilizing organs from DCD donors because they were considered to be of poorer quality; however, an increasing body of evidence now suggests that these concerns are, in part, unfounded, provided DCD organs are transplanted in a timely fashion.^22^ In 2019, over 100 people received a kidney transplant from the living kidney sharing scheme.^23^

The most recent strategy to increase deceased donor numbers involves new legislation that changed England from an “opt in” consent system, in which individuals choose to join the organ donor register, to an “opt out” or deemed consent system, in which individuals are presumed to consent to donating their organs after death, unless they have explicitly specified otherwise.^24^ The Organ Donation (Deemed Consent) Act came into force on May 20, 2020. Data from 22 other countries have demonstrated an increase in deceased organ donation rates as a result of deemed consent.^25^

Living donation provides another source of kidneys for transplantation.^7^ A living donor kidney transplant is considered the “gold standard” treatment for end-stage renal disease because these kidneys are shown to have the best transplant outcome in most circumstances.^26^ In the first decade of the millennium, living donor kidney transplantation activity trebled across the United Kingdom because of the excellent outcomes associated with it and the ability for patients to receive a planned transplant and potentially avoid dialysis. In recent years, living kidney donor numbers have remained stable until the SARS-CoV-2 pandemic, with 1039 people donating in 2019/2020 and 422 in 2020/2021.^27,28^ Living donors may choose to donate to someone they know (“specified” or “directed” donation) or to someone they do not know (“unspecified” or “nondirected” donation).^12,29^

Specified donors represent the vast majority of living kidney donors in the United Kingdom (91%).^29^ The practice of unspecified kidney donation (UKD) is relatively new. UKD, which involves an individual undergoing major surgery for the benefit of someone they have never met, has posed a number of ethical challenges for the transplant community.

Initially, organs from UKDs were offered to an individual on the national transplant waiting list in accordance with UK kidney offering criteria for deceased donation. From 2012, UKDs were given the option to donate into the UK Living Kidney Sharing Scheme (UKLKSS) to initiate a chain of transplants, and since January 2018, this has become the default position. This allocation strategy aims to maximize the benefit gained from these donations. The UKLKSS aims to facilitate compatible living donor transplants between incompatible donor-recipient pairs or an improved age or human leukocyte antigen match between compatible pairs. UKDs have been integrated into this scheme to trigger donor chains (altruistic donor chains), whereby their kidney is allocated to a recipient registered into the scheme and, in turn, the donor registered with that recipient donates to another recipient, and so on. The chain ends when the last donor donates to someone on the national transplant waiting list. If a high-priority recipient is identified as matching with an unspecified donor, the kidney will be offered to that individual before inclusion into the UKLKSS.^30^ A recipient may be considered high priority if they possess characteristics that may make it very difficult for them to receive a kidney as a part of the deceased donor program.

## ECONOMICS AND KIDNEY DONATION

The previous sections have reiterated that there are supply and demand issues around the availability of kidneys for transplantation. A key component of the discipline of economics is to analyze and understand markets applying concepts of “supply” and “demand” to goods and services and observing changes in market behavior due to changes in price. That is not to say that economists are necessarily proponents of markets. Indeed, their study often reveals “market failures,” necessitating alternative approaches. Markets for kidneys and other organs in which payment takes place are illegal in the United Kingdom and across most of the world. Before donating an organ in the United Kingdom, individuals must convince the explanting surgeon that they are doing so on a voluntary basis, without coercion, and in the absence of payment or other financial reward (although the reimbursement of expenses is permitted).^31^ Although illegal in the United Kingdom, it can be argued that examining payment from an economics perspective is still relevant. Payment to living donors does have its advocates, and it is important to understand the arguments that are made in its favor and, when appropriate, to critique these. Payment is a form of “reward,” and it is important to recognize that not all rewards are financial. Living donation, to be discussed later, has rewards for some even when nonspecified.

Arguably, introducing some form of payment to living kidney donors provides a simple solution for the supply problem for kidney transplantation. However, most would consider such a market objectionable. The term “repugnant markets” is applied to transactions between 2 willing parties that are considered objectionable by the society to which they belong. Repugnant markets are therefore restricted or prohibited outright. Roth^32^ argues that what is objectionable is hard to predict and often dependent on the local culture.

Roth^32^ goes on to investigate the kidney trade and suggests that removing a kidney from a living human is itself a potentially objectionable act from the position of a doctor given the Hippocratic principle to “first do no harm.” This applies whether or not there is payment for the kidney being transplanted. It is undeniable that there is near universal opposition to paying people for a kidney. A recent report by the British Broadcasting Corporation highlighted the practice in several countries (including the United States, Canada, Austria, and Germany) of paying people for blood products to meet unmet demand.^33^ Indeed, plasma products from paid blood donors in the United States have been used for many years in the United Kingdom as a result of the ban imposed on UK plasma following the Creutzfeldt-Jakob’s disease crisis. An argument against payment for tissue donation is that donors with low incomes would be disproportionately represented. The World Health Organization’s guiding principles for cell, tissue, and organ transplantation advice against payment for human body parts to prevent exploitation of vulnerable people.^34^

Becker and Elias^35^ have argued that the supply of organs would change from inelastic to highly elastic if payments were made for kidneys, because of the large number of those potentially willing to provide organs and the relatively small number of potential transplant recipients. When the price elasticity of supply is inelastic, the increase in price does not change the quantity supplied by the same proportion. Conversely, when it is highly elastic, the number of kidneys supplied would increase at a greater rate if the price increased.

Becker and Elias^35^ argue for the use of a market system with payment in kidney transplantation and respond to the 3 objections posed by Roth.^32^ They write that first, poorer workers do more dangerous jobs and that this situation (which could also be regarded as exploitation) seems to be tolerated, thereby suggesting double standards. This is a relatively weak argument: further exploiting poorer people for their organs compounds the injustice of exploiting them for their labor. Second, they argue transactions should improve the welfare of both parties, which could occur in the selling of a kidney. This argument can be contested though, and there is little evidence that benefits do ultimately occur for the paid donor. From an ethical perspective, we should consider power and resource imbalances between the parties in the transaction being unequal from the start. Third, Becker and Elias^35^ suggest that the market for kidneys could be regulated to prevent abuse of those with fewer material resources. This is also problematic from an economic perspective because of the cost of regulation and potential for gaming. Although the study of Becker and Elias^35^ is interesting and bold because it estimates a price, trade remains at odds with the views of the vast majority of clinicians and others involved in the provision and evaluation of healthcare. It is worth also pointing out that there is little evidence to suggest that these ideas have support within the economics community either.

Nonetheless, there are examples of markets for organs being established. For instance, kidneys can be legally bought and sold in Iran.^22,23,36,37^ The Iranian model results in most donations being from living instead of deceased kidney donors. Reports have indicated that 60% of Iranians donating a kidney for payment report bad health outcomes after transplant,^35^ suggesting a lack of postoperative care. Deceased donors are responsible for 12% of the kidneys donated in Iran, and similarly, 12% of living donor kidneys are donated to a recipient known to the donor. The remaining transplants involve parties who are unknown to each other and in which payment is made for the organ. These proportions are almost the opposite of the UK system in which 70% of donations are deceased, and of living donors, over 90% are between individuals who are known to one another. In Iran, there are no waiting lists, and over 50% of patients with end-stage renal disease are living with a functioning graft. One could argue that given the high proportion of living donors who are living with bad health after their donation, that the health burden is being shifted from one place to another, and there is a real chance that individuals donating a kidney for payment may themselves be in need of a transplant in the future. The societal impact of this is also unclear. The case of Iran shows that a market for kidneys is feasible, if not without challenges. Whether or not such a market can function, the ethical objections to markets for organs are undiminished.

## PROPOSED SOLUTIONS FOR THE SUPPLY AND DEMAND IMBALANCE IN KIDNEY DONATION

In UKD, there is no pre-existing relationship between the organ donor and the recipient. We assume (when no payment is made for the organ) that the donation is made for altruistic reasons and donors themselves cite the desire to help others as the main motivating factor.^38^ We propose 3 types of solutions for the supply and demand imbalance in kidney donation:

• increasing supply (tax incentives, prioritization of registered donors, reciprocity, and education);
• improving allocation of kidney supply to meet the demand (allocation and collaboration between centers);
• decreasing demand (improved dialysis treatments and kidney disease prevention).

### Increasing Supply

#### Tax Incentives for Deceased Donation

Bilgel and Galle^39^ describe how certain states in New York have introduced tax incentives for people who register for organ donation after their death. They apply a number of complex models, and the results suggest that there may be an increase in donations from deceased donors, (incentivizing people to join the transplant list) if tax incentives were offered.

#### Prioritization of Registered Donors as Recipients of a Transplant

Stoler et al^40^ illustrate that priority transplantation for people who have previously agreed to be an organ donor after their death is a powerful incentive for joining the deceased donor register based on experiences in Israel. Before 2008, it was thought that people were wanting to receive deceased donor organs but not donate them (free riding or negative reciprocity).^41^ The Israeli Organ Transplantation Law of 2008 increased the number of people joining the list of deceased organ donors. The deadline for joining the list, after which there would be a delay in gaining priority, increased the number of people joining still further, even though there was a fall in registrations after the deadline. As with the case of the tax incentive in New York, there may be a benefit to introducing this nonpayment incentive to join the organ donation register. Programs that aim to educate the public about organ donation, like those in the NHS currently,^42^ may also increase the amount of living donors as many more people become aware of the positive impact this can have.

### Improving Allocation of Kidney Supply to Meet the Demand

#### Kidney Exchanges and Kidney Chains

As mentioned above, the UKLKSS facilitates transplants between incompatible pairs or a chain of transplants by the introduction of an unspecified donor. The first UK kidney exchange between 2 pairs took place in 2007.^43^ Sönmez and Ünver^44^ discuss kidney exchanges within a scheme such as the UKLKSS and note that although a great deal has been achieved through these systems in several countries, there are 3 areas in which they can improve: (1) by including compatible pairs (ie, those who could be transplanted out with the scheme) so that pairs with greater compatibility can be found; (2) by increasing the size of the pool for kidney exchanges; and (3) by using dynamic matching in which the matching process is seen as occurring over time and donations and transplants are staggered, rather than being conducted simultaneously.

They also suggest that transplant centers should collaborate (as they do in the United Kingdom), although this might be a suboptimal strategy for individual transplant centers that may find they achieve fewer matches individually, even though more transplant operations are made as a collective. A center may, for example, achieve fewer matches if a kidney donor from their center is a better match for someone in another center rather than their own. This is problematic if an internal market between centers is used to determine funding. More recently, Biró et al^45^ suggest that kidney exchange programs can be improved in 3 main ways: (1) by extending national programs to include all transplant centers, (2) by allowing for different and novel modalities in the exchanges, and (3) increased international cooperation between transplant centers. The second and third of these are already happening in the United Kingdom. Finally, kidneys from UKDs can be used to “prime” a chain of transplants within a kidney exchange program, with the final kidney in the chain going to someone on the national transplant waiting list. This has been highly successful in the United Kingdom.^30^ For example, in 2018/2019, 64 UKDs donated into the sharing scheme, priming living donor chains, which resulted in 134 transplants.^17^

#### Increasing Living Donation

In the early 2000s, a significant resource injection of £5 million was made to increase living kidney donation (beyond previous limits of 1 or 2 centers who had appointed a dedicated living donor coordinator) and resulted in a trebling of living kidney donor numbers. As yet, we do not know whether this was a cost-effective use of resources. Further strategies to increase numbers may include increasing general awareness of the potential to save someone’s life through donating a kidney.^46^ The idea of encouraging UKD is subject to debate because of ethical considerations.^47^ Glannon^48^ argues that a doctor who the patient trusts because of greater knowledge and experience should not encourage a patient to subject themselves to risk of poorer health outcomes to improve the health of another patient. Williams^49^ states that the donation of a kidney is likely to be acceptable if 3 conditions are met. First, the donor gives valid consent; second, there is an overall balance of benefit for donor and recipient; and third, donation is unlikely to result in significant morbidity or death for the donor. Living donation (specified or not) is interesting from an economic perspective because the cost-effectiveness has not yet been established. It makes sense that utilization of kidneys for high-priority recipients (who are likely to wait for a long time on the national deceased donor waiting list) and their use in the UKLKSS is reducing the financial burden on the health service. One of the aims of the BOUnD study is to conduct an economic analysis of the monetary value of an unspecified donor kidney alongside the of transplantation, donor healthcare service use, and quality of life.^12^

### Decreasing Demand

This article has focused on the fact that the demand for kidneys outstrips their supply. We have emphasized the limitations of conventional markets (in which a supplier receives compensation) to increase supply. In addition to concerns about efficiency, economics is also concerned with improving an equitable distribution of resources, and approaches to address such inequities could be an important aspect of kidney exchange programs. The demand sides should also be emphasized. Although a long way off, avoidance of renal failure in the first place would also reduce demand for kidneys.

### Improved Dialysis, Kidney Disease Prevention, and Posttransplant Care

A potential option to reducing the demand for kidneys is to improve the effectiveness and reduce the cost of dialysis so that it becomes a more clinically effective and cost-effective treatment option in comparison to transplantation. Current dialysis methods, however, have no clear evidence of superiority over transplantation. Reducing the incidence of ESKD is not only a reasonable strategy but a complex one. Modifiable risk factors for ESKD include obesity, hypertension, and diabetes. This is a multifaceted challenge and requires effective public health interventions that encourage healthier lifestyles through behavior change and maintenance. Increasing research into medications that are effective in the earlier stages of CKD and may slow progression and need for renal replacement therapy (dialysis and transplant) is another potential solution. Furthermore, interventions designed to better support self-management behaviors (including medication adherence) in those with CKD may also attenuate or even prevent disease progression. Managing the care of patients’ post transplantation is also important to prevent the need for retransplantation and malignancies.^50,51^

## CONCLUSIONS

The number of people living with ESKD is growing, and this places a considerable burden on the resources of the NHS because of the cost of renal replacement therapy, which is costly in comparison to transplantation. Although living kidney donation provides the best quality organs for transplantation, leading to better outcomes for patients, using market forces to encourage trade in kidneys to reduce the gap between supply and demand is generally considered morally repugnant. It is therefore important to either find alternative solutions to the lack of supply of kidneys for transplantation or reduce the need for kidney donation and develop more effective methods for treating end-stage renal disease. This article has explored the potential for a market for kidneys and has presented 4 policy approaches that may resolve what can be seen, from an economic perspective, as a supply and demand problem in kidney transplantation.
